# Supplementary material for: The Antibiofilm Effects of Antimony Tin Oxide Nanoparticles against Polymicrobial Biofilms of Uropathogenic Escherichia coli and Staphylococcus aureus
Source: Pharmaceutics. 2023 Jun 8;15(6):1679. doi: 10.3390/pharmaceutics15061679 (PMC10301033; doi:10.3390/pharmaceutics15061679)
Supplement: Supplementary file 1 [file pharmaceutics-15-01679-s001.zip › pharmaceutics-2397398-supplementary.pdf]

pharmaceutics

Supplementary Information
The antibiofilm effects of antimony tin oxide nanoparticles against polymicrobial biofilms of uropathogenic *Escherichia coli* and *Staphylococcus aureus*

Inji Park†, Afreen Jailani†, Jin-Hyung Lee, Bilal Ahmed, Jintae Lee*

Table S1. Sequences of the primers used for quantitative RT-PCR.

Gene	Name	Primer sequence
agrA	Quorum-sensing regulator A	Forward 5'-TGA TAA TCC TTA TGA GGT GCT T-3'<br>Reverse 5'-CAC TGT GAC TCG TAA CGA AAA-3'
aur	Zinc metalloproteinase aureolysin	Forward 5'-ACC GTG TGT TAA TTC GTG TGC TA-3'<br>Reverse 5'-ATG GTC GCA CAT TCA CAA GTT T-3'
hla	α-Hemolysin	Forward 5'-CGG CAC ATT TGC ACC AAT AAG GC-3'<br>Reverse 5'-GGT TTA GCC TGG CCT TCA GC-3'
icaA	Intercellular adhesion A	Forward 5′-TGA ACC GCT TGC CAT GTG-3′<br>Reverse 5′-CAC GCG TTG CTT CCA AAG A-3′
nuc1	Nuclease	Forward 5'-CAC CTG AAA CAA AGC ATC CTA A-3'<br>Reverse 5'-TAT ACG CTA AGC CAC GTC CAT-3'
RNAⅢ	Transcriptional regulator	Forward 5'-ATC GAC ACA GTG AAC AAA TTC AC-3'<br>Reverse 5'-CTC TAC TAG CAA ATG TTA CTC AC-3'
saeR	Response regulator	Forward 5'-GCC TTA ACT TTA GGT GCA GAT GAC TAT GTC-3'<br>Reverse 5'-CGA CAG TTG TTC AAC TGG TTG ATG ATG G-3'
sarA	Transcriptional regulator	Forward 5'-GAG TTG TTA TCA ATG GTC-3'<br>Reverse 5'-GTT TGC TTC AGT GAT TCG-3'
seb	Enterotoxin B	Forward 5'-TGT TCG GGT ATT TGA AGA TGG -3'<br>Reverse 5'-CGT TTC ATA AGG CGA GTT GTT-3'
sigB	RNA Polymerase sigma factor	Forward 5'-AAG TGA TTC GTA AGG ACG TCT-3'<br>Reverse 5'-TCG ATA ACT ATA ACC AAA GCC T-3'
spa	Protein A	Forward 5'-ACC AGA AAC TGG TGA AGA AAA TCC-3'<br>Reverse 5'-TAA CGC TGC ACC TAA GGC TAA TG-3'
16S rRNA	A component of ribosomes	Forward 5'-TGT TTG ACG ATG TTT GAG CA-3'<br>Reverse 5'-CCT TCC TCC AGT TCA GAT GC -3'

Table S2. The characteristics of antimony tin oxide (ATO) nanoparticles provided by Sigma-Aldrich

Size	>50 nm
Density	5.2 g/cm^3
Melting point	655 °C
Tin dioxide composition	90 ~ 95%
Diantimony pentoxide composition	10 ~ 15%

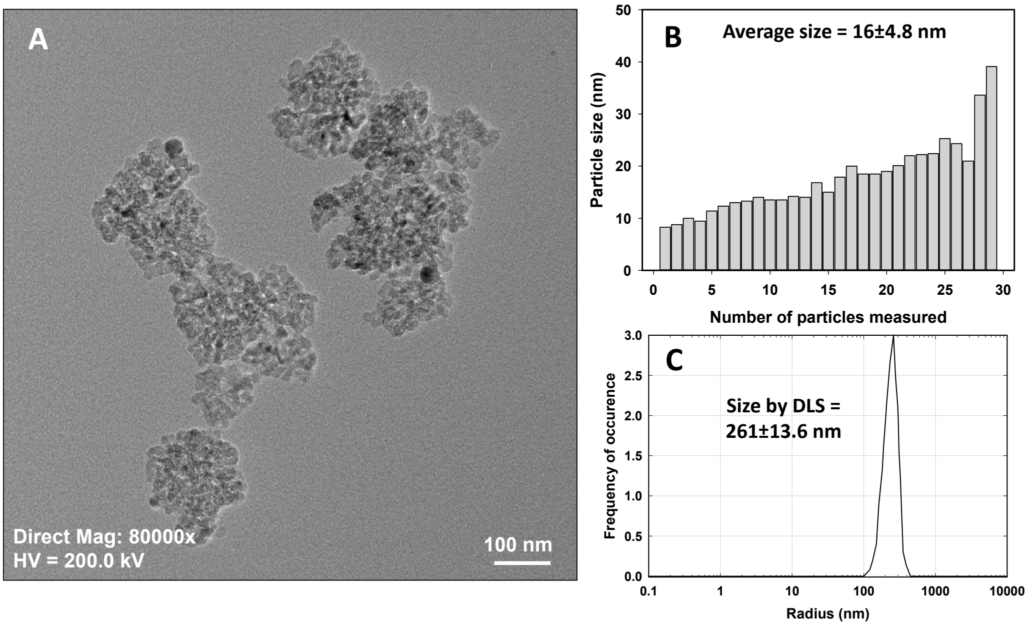

Figure S1. Characterization of ATO NPs by TEM (A). Panel B shows average size by TEM and panel C is for hydrodynamic size by DLS.

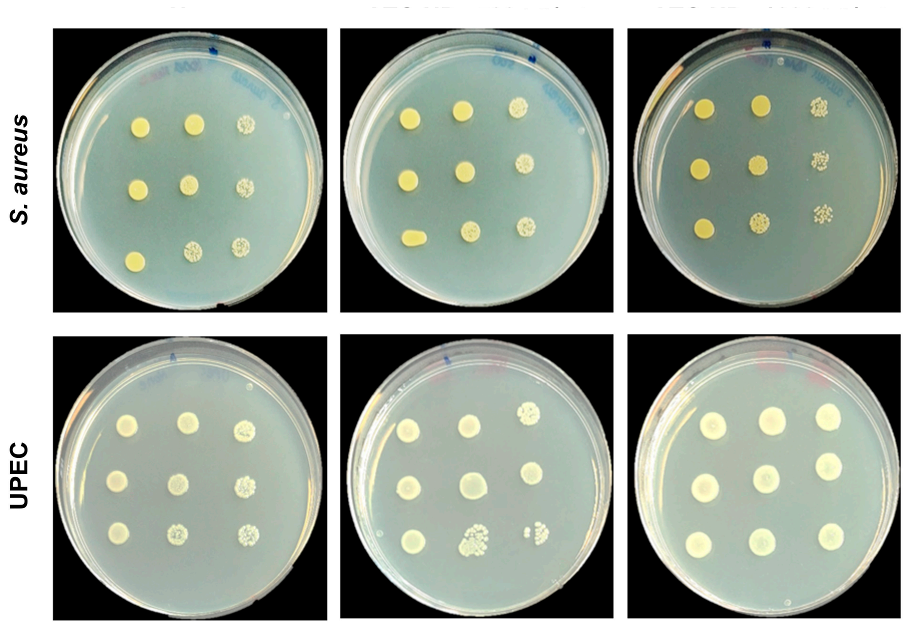

Figure S2. Antimicrobial activity of ATO NPs against *S. aureus* and UPEC.

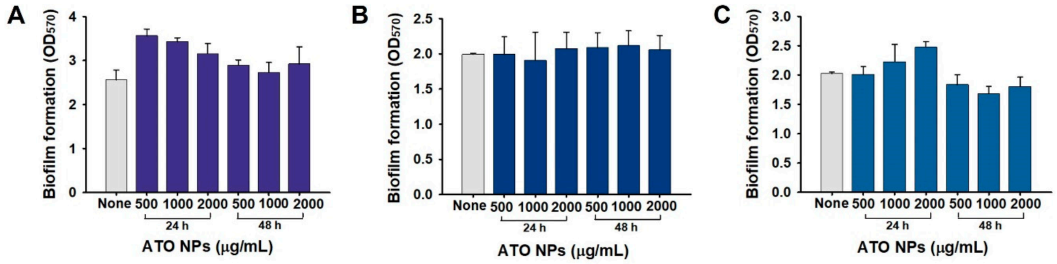

Figure S3. Biofilm dispersal ability of ATO NPs against *S. aureus* (A), UPEC (B) and *S. aureus*/UPEC (C). None indicates untreated biofilm formation for 24 h.

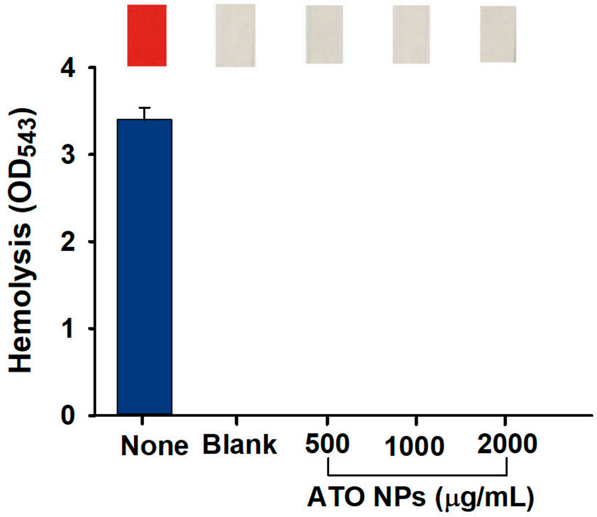

Figure S4. The effects of ATO NPs alone on blood hemolysis. None indicates the presence of *S. aureus* without ATO NPs. ATO NPs indicate the addition of ATO NPs without bacteria.
